# Aloe vera for treating acute and chronic wounds

**DOI:** 10.1590/1516-3180.20141326T1

**Published:** 2014-09-02

**Authors:** Anthony D. Dat, Flora Poon, Kim B. T. Pham, Jenny Doust

## Abstract

**BACKGROUND:**

Aloe vera is a cactus-like perennial succulent belonging to the Liliaceae Family that is commonly grown in tropical climates. Animal studies have suggested that Aloe vera may help accelerate the wound healing process.

**OBJECTIVE:**

To determine the effects of Aloe vera-derived products (for example dressings and topical gels) on the healing of acute wounds (for example lacerations, surgical incisions and burns) and chronic wounds (for example infected wounds, arterial and venous ulcers).

**METHODS:**

*Search methods:* We searched the Cochrane Wounds Group Specialised Register (9 September 2011), the Cochrane Central Register of Controlled Trials (CENTRAL) (The Cochrane Library 2011, Issue 3), Ovid MEDLINE (2005 to August Week 5 2011), Ovid MEDLINE (In-Process & Other Non-Indexed Citations 8 September 2011), Ovid EMBASE (2007 to 2010 Week 35), Ovid AMED (1985 to September 2011) and EBSCO CINAHL (1982 to 9 September 2011). We did not apply date or language restrictions. *Selection criteria:* We included all randomised controlled trials that evaluated the effectiveness of Aloe vera, aloe-derived products and a combination of Aloe vera and other dressings as a treatment for acute or chronic wounds. There was no restriction in terms of source, date of publication or language. An objective measure of wound healing (either proportion of completely healed wounds or time to complete healing) was the primary endpoint. *Data collection and analysis:* Two review authors independently carried out trial selection, data extraction and risk of bias assessment, checked by a third review author.

**MAIN RESULTS:**

Seven trials were eligible for inclusion, comprising a total of 347 participants. Five trials in people with acute wounds evaluated the effects of Aloe vera on burns, haemorrhoidectomy patients and skin biopsies. Aloe vera mucilage did not increase burn healing compared with silver sulfadiazine (risk ratio (RR) 1.41, 95% conﬁdence interval (CI) 0.70 to 2.85). A reduction in healing time with Aloe vera was noted after haemorrhoidectomy (RR 16.33 days, 95% CI 3.46 to 77.15) and there was no difference in the proportion of patients completely healed at follow up after skin biopsies. In people with chronic wounds, one trial found no statistically signiﬁcant difference in pressure ulcer healing with Aloe vera (RR 0.10, 95% CI -1.59 to 1.79) and in a trial of surgical wounds healing by secondary intention Aloe vera signiﬁcantly delayed healing (mean difference 30 days, 95% CI 7.59 to 52.41). Clinical heterogeneity precluded meta-analysis. The poor quality of the included trials indicates that the trial results must be viewed with extreme caution as they have a high risk of bias.

**AUTHORS' CONCLUSIONS:**

There is currently an absence of high quality clinical trial evidence to support the use of Aloe vera topical agents or Aloe vera dressings as treatments for acute and chronic wounds

**This is the abstract of a Cochrane Review published in the Cochrane Database of Systematic Reviews (CDSR) 2012, issue 2. Art. No.:** CD008762. DOI: 10.1002/14651858.CD008762.pub2 (http://onlinelibrary.wiley.com/doi/10.1002/14651858.CD008762.pub2/abstract). For full citation and authors' details see reference 1.

**The full text of this review is freely available from: **http://onlinelibrary.wiley.com/doi/10.1002/14651858.CD008762.pub2/pdf.

**The abstract is available from:** http://summaries.cochrane.org/CD008762/WOUNDS_aloe-vera-for-treating-acute-and-chronic-wounds.
